# Supplementary material for: Tunable plasmonic resonances in Si-Au slanted columnar heterostructure thin films
Source: Sci Rep. 2019 Jan 11;9:71. doi: 10.1038/s41598-018-37153-x (PMC6329775; doi:10.1038/s41598-018-37153-x)
Supplement: Supplementary file 1 — Comparison GSE vs FEM result: Si-Au SCHTF thickness evolution [file 41598_2018_37153_MOESM1_ESM.pdf]

Supplementary material:Tunable plasmonic resonances in Si-Au slanted columnar heterostructure thin films

Ufuk Kılıç[1,*], Alyssa Mock[1,2], René Feder[3], Derek Sekora[1], Matthew Hilfiker[1], Rafał Korlacki[1], Eva Schubert[1], Christos Argyropoulos[1], Mathias Schubert[1,2,4]
[1]Department of Electrical and Computer Engineering, University of Nebraska-Lincoln, Lincoln, NE 68588, USA
[2]Department of Physics, Chemistry, and Biology, Linköping University, 58183 Linköping, Sweden
[3]Fraunhofer Institute for Microstructure of Materials and Systems (IMWS), D-06120, Halle (Saale), Germany
[4]Leibniz Institute for Polymer Research, Dresden, D-01005, Germany
[1]ufuk.kilic@huskers.unl.edu, christos.argyropoulos@unl.edu, URL: http://ellipsometry.unl.edu

Comparison GSE vs FEM result: Si-Au SCHTF thickness evolution

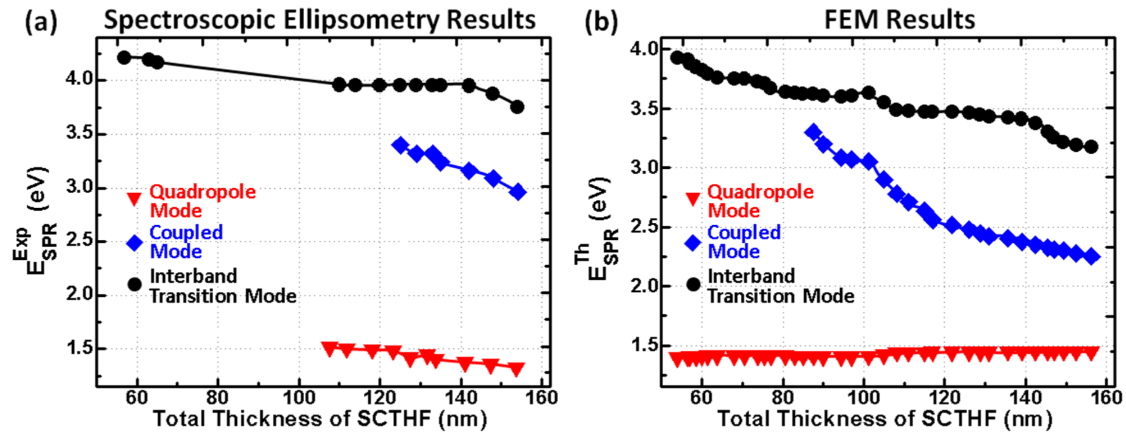

Fig S1 Spectral positions of modes identified by GSE analysis (a) and by FEM analysis (b) from the spectral peak behavior of the extinction coefficient for electric field direction parallel to the Si-Au SCHTF column axis, as a function of total Si-Au SCHTF thickness. The ratio between Si and Au subcolumn length in all samples is constant and approximately 12.8.

Figures S1(a), and (b) show the spectral positions of the three modes identified in Figure 5(a) and (b) by ellipsometry analysis, and by computational modeling analysis, respectively, from the spectral peak behavior of the extinction coefficient for electric field direction parallel to the column axis, as a function of total Si-Au SCHTF thickness. An excellent agreement can be seen between the results from our experimental and computational model approaches. The quadrupole-like mode reveals very little, if any, dispersion with increasing thickness in the computational result while a small red-shift is seen from the experiment. We assign this slight disagreement to small structural

disorder in the real SCHTF structures versus the assumed perfect geometry in the FEM calculations. During our computational efforts we noted that the quadrupole-like mode is very sensitive to the ratio between the Si and Au subcolumn lengths, hence, to the Si-Au volume ratio. It is for this reason that the ratio was kept constant during the GLAD deposition process. Small variations in Si-Au volume ratio cause subtle shifts of the quadrupole-like mode to lower or higher photon energies, and which thereby can be used as parameter to tune the quadrupole-like mode frequency. The absolute values of the mode energies are slightly different between ellipsometry and FEM results, for example, the inter-band transition-like mode is red shifted overall in the FWM result. This discrepancy may be due to the fact that the spectral dependence of the dielectric constants measured from non-porous polycrystalline Si thin films were used as FEM input parameter for the Si subcolumns, while their true dielectric functions may be slightly different.
